# Transgenerational transmission of psychopathology: when are adaptive emotion regulation strategies protective in children?

**DOI:** 10.1186/s13034-024-00783-3

**Published:** 2024-08-07

**Authors:** Arleta A. Luczejko, Naomi Leona Werkmann, K. Hagelweide, R. Stark, S. Weigelt, H. Christiansen, M. Kieser, K. Otto, C. Reck, R. Steinmayr, L. Wirthwein, A.-L. Zietlow, C. Schwenck, the COMPARE-family research group

**Affiliations:** 1 Department of Clinical Child and Adolescent Psychology, Justus Liebig University Giessen, Otto-Behaghel-Str. 10, 35394 Giessen, Giessen, Germany; 2 Department of Psychotherapy and Systems Neuroscience, Justus Liebig University Giessen, Giessen, Germany; 3 Department of Rehabilitation Sciences, Technical University Dortmund, Dortmund, Germany; 4 Department of Psychology, Clinical Child and Adolescent Psychology, Philipps University Marburg, Marburg, Germany; 5 Institute of Medical Biometry, University of Heidelberg, Heidelberg, Germany; 6 Department of Work and Organizational Psychology, Philipps-University Marburg, Marburg, Germany; 7 Department of Psychology, Ludwig-Maximilians-Universität München, Munich, Germany; 8 Department of Psychology, Technical University Dortmund, Dortmund, Germany; 9 Clinical Child and Adolescent Psychology, Department of Psychology, Technische Universität Dresden, Dresden, Germany

**Keywords:** Transgenerational transmission of mental disorders, Emotion regulation, Adaptive emotion regulation strategies, Maladaptive emotion regulation strategies, Psychopathology, Parental mental illness, Prevention

## Abstract

**Background:**

Children of parents with a mental illness (COPMI) have multiple psychological and developmental risks, including an increased lifetime risk of developing a mental illness themselves. Emotion regulation (ER) has been identified as a potential underlying mechanism of the transgenerational transmission of mental disorders. This study compares ER strategies in parents with and without a mental illness and their children. Further, it aims to examine the relationship between parents and children’s psychopathology with a focus on the role of parental and child ER.

**Methods:**

Participants were 96 COPMI (77% female) and 99 children of parents without mental illness (COPWMI, 83% female) aged 4–16 years and their parents. Psychopathology and ER strategies of parents and children were assessed with a series of questionnaires.

**Results:**

Both COPMI and their parents showed significantly more psychopathology and more maladaptive and adaptive ER strategies in comparison with COPWMI and their parents. Parent and child adaptive ER strategies mediated the relationship between the psychopathology of parents and children only when child maladaptive ER strategies were low.

**Conclusions:**

The findings further our understanding of the processes by which parental psychopathology affects child outcomes. Our findings highlight the importance of implementing preventive programs that specifically target the reduction of maladaptive ER in children to interrupt the transgenerational transmission of psychopathological symptoms.

## Background

### Children of parents with a mental illness

 Children of parents with a mental illness (COPMI) are most likely to be the next generation of patients with a mental illness [1, 2]. According to estimates, approximately 25% of children live in a household with at least one mentally ill parent at some time [3–6]. Compared to children of parents without mental illness (COPWMI), COPMI have multiple psychological and developmental risks. In particular, COPMI do not only show more subclinical internalizing and externalizing symptoms [7, 8] but also an increased lifetime risk to develop a mental illness themselves [2, 9, 10]. Thus, a transgenerational transmission of mental disorders (TTMD) can be assumed, which makes them a target group for selective prevention programs [2, 11–13].

The TTMD model assumes different transmission mechanisms and their interplay to be responsible for the transmission of mental disorders. These mechanisms are grouped into (a) parent-related factors, such as the impairment and chronicity of the parental disorder, parenting competence, risk- and protective factors, (b) family-related factors (e.g. the socioeconomic status, possible violence and marital discord, factors related to the other parent), (c) child-related factors such as vulnerability, attachment style, cognitive skills and (d) factors related to the social environment (e.g. isolation, social support, availability of professional care). Further, genetic transfer and parent-child interaction are included in the model. Although the impact of the single factors is not sufficiently tested yet, parent- and child-related factors display a promising target for preventive measures. Though not mentioned directly in the model, ER is of particular importance in this context, as it has been shown to be associated with psychopathology in children and adults alike [14–18] and is associated with multiple factors included in the TTMD model [19–25].

### Emotion regulation

ER comprises processes that influence the incidence, kind, intensity, and duration of emotions as well as their effects on feelings and behaviors [26, 27]. It describes a heterogeneous construct that includes physiological, cognitive, and behavioral facets [28]. One facet of ER is use of ER strategies, which summarize all strategies used to influence emotions and their effects on behaviors. ER strategies can be classified in multiple ways. One of the most common ways of classifying ER strategies is categorizing them into adaptive and maladaptive strategies. According to the literature, adaptive strategies are defined by being associated with positive long-term consequences (e.g., well-being and reduction of psychopathological symptoms), while maladaptive ER strategies increase negative long-term consequences such as psychopathological symptoms and negative life outcomes [29–31]. Common ER strategies classified as adaptive include cognitive re-appraisal, problem-solving, acceptance and distraction, while rumination or suppression are typically classified as maladaptive [32].

ER development starts in infancy and carries on throughout childhood into early adulthood and is influenced by genetic as well as environmental factors [33]. In infancy, ER has been found to be an almost exclusively interpersonal process mainly within parent-child dyads [25, 33–35]. It is crucial for caregivers to correctly interpret children’s emotional cues and respond appropriately to form a secure attachment [36] and to lay the foundation for children’s own ER abilities [21]. Throughout early childhood parents can build on this foundation as they continue to have a significant impact on their child’s acquisition and application of ER strategies via emotion socialization, which involves explicit as well as implicit behaviors teaching children how to understand and regulate their emotions, e.g. modelling emotions and emotional responses, parenting behaviors and family climate [21, 37–43]. Studies examining the relationship between parental and children’s ER strategies provide evidence, suggesting that parents’ and children’s use of adaptive as well as maladaptive ER strategies is related in a bidirectional manner [43–47]. A second factor implicitly influencing children’s ER abilities is the parent-child attachment. Since parents influence ER and attachment through similar behaviors in infancy, it is unsurprising that ER and attachment are closely related later on in life. Multiple studies have confirmed this, suggesting a bidirectional relationship with secure attachment and higher ER skills being connected throughout childhood into adolescence [48–52]. Here, not only mothers play a role: multiple studies as well as literature reviews suggest that fathers and mothers both influence children’s ER in the context of attachment [48, 51], with some studies suggesting that mothers and fathers influence children’s ER abilities equally but with different profiles [53]. During adolescence, ER abilities undergo significant changes due to changes in internal factors influencing ER, such as an increase in cognitive abilities [54] as well as emotional reactivity [55, 56]. Further, external factors also seem to play a role in this process. While ER during infancy and childhood is mainly influenced by parents, during adolescence the influence of parents on their child decreases while the influence of others (e.g. peer-group) increases [57, 58]. In their review, Zimmer-Gembeck et al. [59] summarized two trends for ER strategy use in adolescence: For one, regulatory capacities seem to increase compared to childhood, which is reflected in a more sophisticated repertoire of ER strategies and an increased understanding of emotional situations. Secondly, there seems to be an improvement in tailoring regulatory attempts to a situation, meaning that adolescents pick the most efficient strategy for a specific situation rather than picking at random [59].

### The role of ER in the TTMD

Less frequent use of adaptive and more frequent use of maladaptive ER strategies is accompanied by increased psychopathological symptoms in both children and adults [60–63]. Although the empirical data on ER in COPMI is limited, it confirms the relevance of ER for the TTMD. A study of young children (ages 4–7) of mothers with either depression or without depressive symptomatology compared ER strategies between these two groups. The results indicate more maladaptive ER in children of mothers with a depression diagnosis compared to the other group [64]. A study comparing ER in children (age 4) of mothers with a history of childhood-onset depression and with no history of mental disorder found similar results. Compared to children of mothers without a depression history, children of mothers with past depressive disorder were more likely to exhibit maladaptive versus adaptive ER [65]. These cross-sectional studies are complemented by a longitudinal study showing that maternal depression (during the first 21 months of the child’s life) predicted dysregulated emotion patterns in children at age 4 [66]. The latest study on the subject expands the findings described so far. Children of depressed and non-depressed parents were compared inter alia regarding ER and psychopathology. In comparison with children of non-depressed parents, children of depressed parents showed more symptoms of depression and general psychopathology. Furthermore, children of depressed parents showed less adaptive ER strategies. Although the groups did not differ in maladaptive ER strategies, maladaptive ER strategies partially mediated the association between parental depression and children’s depressive symptoms [7].

In addition to studies with children of parents with depression diagnoses, investigations in (high-risk) community samples seem to confirm associations between parental psychopathology, ER of children or parents and psychopathology of children [67–69]. ER difficulties in children (ages 9–14) mediated the relationship between maternal personality disorder symptoms and child behavior problems one year later [67]. While the studies described so far relate to the ER of children, Han et al. [68] Kerns et al. and [69] consider parental ER. Han et al. (2016) identified parental emotion dysregulation as a mediator of the relationship between parental psychopathological symptoms and child internalizing problems. Kerns et al. (2017) tested a sequential mediation model and found that maternal anxiety predicted maladaptive maternal ER. Maternal maladaptive ER predicted greater maternal accommodation, which, in turn, predicted higher child anxiety.

Previous studies have largely examined the relation of ER and psychopathology separately for adaptive and maladaptive ER strategies [14, 61]. A meta-analysis compared effect sizes and indicated that elevated maladaptive ER strategies were more strongly associated with higher psychopathology than reduced adaptive ER strategies [14]. However, recent research has emphasized the importance of examining the interaction between these two in predicting psychopathology to understand when adaptive ER strategies are more or less useful [29]. Aldoa and Nolen-Hoeksema (2012) propose two hypotheses regarding how maladaptive strategies might moderate the relationship between adaptive ER strategies and psychopathology. On the one hand, maladaptive ER strategies may interfere with using adaptive ER strategies (interference hypothesis, [29]). Thus, adaptive ER strategies can be more difficult to use for people who frequently use maladaptive ER strategies, leading to higher psychopathology [29]. On the other hand, adaptive ER strategies may compensate for the use of maladaptive ER strategies and prevent psychopathology (compensatory hypothesis, [29]). In consequence, adaptive ER strategies may be most important for people frequently using maladaptive ER strategies [29]. Analysis of non-clinical samples of adults support the compensatory hypothesis [29, 70, 71]. However, there is also evidence that did not find a significant interaction between adaptive and maladaptive ER strategies [72]. To the best of our knowledge, there are no studies that have examined this interaction in children or adolescents.

In sum, there is a lack of studies investigating the relationship between ER and psychopathology in COPMI. No study directly investigated the (sequential) mediating role of parental as well as children’s ER strategies on the relationship between psychopathology of parents and children although separate relationships have been examined and confirmed. Interactive effects of adaptive and maladaptive ER also have never been the subject of research in children. Identifying the mechanisms of risk is of clinical importance since adverse patterns of ER strategies can be targeted in preventive interventions and buffer the impact of parental mental illness on children. To develop effective ER training, we need to understand when adaptive ER strategies are more or less protective for the development of psychopathological symptoms.

### The current study

The first aim of the current study is to replicate the finding that COPMI have an increased risk of psychopathology compared to COPWMI. The second aim is to compare ER strategies of parents with and without mental illness. It is hypothesized that parents with mental illness show more maladaptive and fewer adaptive ER strategies than healthy parents. Given the relation of parental and child’s ER strategies, we assume the same pattern for COPMI and COPWMI. Finally, a moderated sequential mediation model is tested. In this context, we expect the following relationships:

1. Parental psychopathology positively predicts child psychopathology (internalizing/externalizing symptoms) [7, 8].
2. Parental psychopathology negatively predicts parental adaptive ER strategies [7].
3. Parental adaptive ER strategies positively predict child adaptive ER strategies [43].
4. The relationship between child adaptive ER strategies and child psychopathology is moderated by child maladaptive ER strategies [29, 70, 71]. Due to the lack of studies on the interaction of adaptive and maladaptive ER strategies in children, two competing options are investigated. First, an interference hypothesis would suggest that adaptive ER strategies would have a weaker negative or no relationship with psychopathology when levels of maladaptive ER strategies are high compared to when they are low. Second, a compensatory hypothesis would suggest that adaptive ER strategies would have a (stronger) negative relationship with psychopathology when levels of maladaptive ER strategies are high than when they are low [29].
5. The relationship between parental psychopathology is indirectly related to child psychopathology (internalizing/externalizing symptoms) through the above-constituted moderated sequential mediation.

**Fig. 1:**
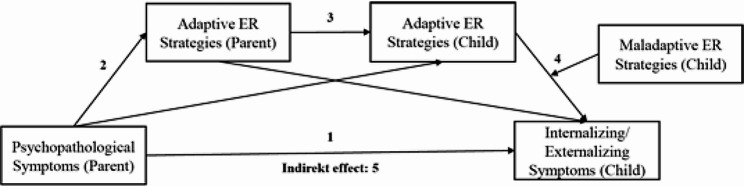
Diagram of sequential moderated mediation model of proposed mediators of the relationship between parental and child psychopathology

Separate models for internalizing and externalizing symptoms are tested (see Fig. 1 for demonstration).

## Methods

The present study is part of the project Children of Mentally Ill Parents At Risk Evaluation and its add-on project COMPARE-Emotion. The projects are described in detail in the study protocols [3, 73].

### Participants

Two hundred parents with mental illness signed informed consent for participating in the add-on project COMPARE-Emotion. However, complete data sets were only available for 125 independent parent-child dyads. Of these dyads, in turn, other information from the COMPARE-Family project such as psychopathology of parents or children were missing (*N* = 29). One hundred parents without mental illness signed informed consent for COMPARE-Emotion. One of these families filled out the questionnaires incompletely. In the end, for the current study complete data sets of *n* = 195 independent parent-child dyads including 96 COPMI and 99 COPWMI were available. Children ranged in age from four to sixteen years (*M* = 9.77, *SD* = 3.16) and included 88 males (45%). COPMI and COPWMI groups did not differ in child age or child gender. Children’s age was evenly distributed across males and females, *t*(193) = 0.65, *p* = .516, Cohen’s *d* = 0.09. Parents of COPMI were younger than parents of COPWMI. Furthermore, the socioeconomic status (SES) of COPMI was lower than of COPWMI. However, looking at representative data of children and adolescent in Germany, the SES of both groups can be categorized as low [74]. For demographic characteristics of participants separately for COPMI and COPWMI see Table 1.

**Table 1:** Demographic characteristics of participants

	COPMI (*N* = 96)	COPWMI (*N* = 99)	t(193)/ χ^2^(1)	*p*	Cohen’s d/φ
**Children**					
Age, *M* (*SD*)	9.34 (3.16)	10.18 (3.13)	1.86	0.064	0.27
Gender (female, %)	55 (57.29)	52 (52.53)	0.45	0.504	0.05
**Parents**					
Age, *M* (*SD*)	40.96 (6.48)	42.90 (6.08)	2.16	0.032	0.31
Gender (female, %)	74 (77.08)	82 (82.82)	1.01	0.316	− 0.07
SES	4.69 (0.99)	6.06 (0.85)	10.33	< 0.001	1.48
BSI GSI (*T* Scores)	61.06 (8.39)	43.12 (7.85)	− 15.42	< 0.0001	− 2.21

SES = Socioeconomic status; BSI = Brief Symptom Inventory, GSI = Global

Severity Index

 50% of mentally ill parents had a Depressive Disorder as primary diagnosis. The number of comorbid diagnoses in parents with mental illness ranged between 0 and 5 (*M* = 1.16, *SD* = 1.16). The average of the severity of the primary diagnosis was six (range from 3 to 8).

### Participant recruitment and study inclusion criteria

COPMI were recruited as part of a randomized controlled multicenter study of a preventive intervention for COPMI in Germany (COMPARE-Family) [3, 73]. The patients were primarily recruited from the University outpatient clinics at each study site. In the study center in Giessen patients were recruited in addition by mailings of randomly picked addresses of families with children in the corresponding age range provided by the local registry office, public advertisement (flyer, newspaper), inpatient psychiatric clinics and the University’s internal mailing list. COPWMI were recruited as part of the add-on project COMPARE-Emotion in addition via the research group’s database of former study participants. Inclusion criteria for COMPI were: (a) between 4 and 16 years of age, (b) parent with a mental illness according to the Diagnostic and Statistical Manual of Mental Disorders (DSM-5) [75]. For COPWMI inclusion criteria were (a) between 4 and 16 years of age, (b) parents without mental disorders and without psychotherapeutic treatment during the past 5 years and after the child was born. Exclusion criteria were (a) insufficient German language skills of children and the parents, (b) severe impairment of the children requiring comprehensive treatment, (c) parental ongoing outpatient or inpatient treatment, or continuous use of benzodiazepines.

The study was approved by the local ethics committee of Faculty 06 of the Justus Liebig University Giessen (number 2017-0050). All participants and their parents gave written informed consent. Parents received an expense allowance of €50 (COPWMI)/€15 (COPMI) for the participation in the add-on project. While the families of the COPWMI group only took part in the add-on project once, the assessment was repeated for the families of the COPMI group at three measurement points according to the clinical study protocol [73]. From the COPMI group the data of the first assessment point of the study were analyzed.

### Measures

#### Outcome measures

##### Psychopathology of parents and children

*Brief Symptom Inventory (BSI).* The mental impairment level in parents of COPMI and COPWMI was assessed using the Global Severity Index (GSI) of the BSI. The BSI is a self-report questionnaire and contains 53 items that are rated on a 5-point Likert scale (0 = “not at all” to 4 = “very much”). Internal consistency has been shown to be very good for the GSI in previous studies (Cronbach’s alpha = 0.97) [76] and the internal consistency found in our study sample aligns with these findings (Cronbach’s alpha = 0.96).

*Child Behavior Checklist (CBCL).* Depending upon the age of the children, we applied the German versions of the parent-report measure CBCL 1½-5 [77] or the CBCL 6-18R [78] from the Achenbach system of empirically based assessment in COMPI and COPWMI. The German version of the CBCL 1½-5 [77] consists of 99 items assessing problems of children between the age of 1,5 and 5 years using a 3-point Likert scale (0 = “not true” to 2 = “very true or often true”). The items constitute three superordinate scales (“externalizing problems”, „internalizing problems“, and „total problems”). Although the German version still requires verification of the reported reliabilities, Achenbach and Rescorla (2000) list test-retest reliabilities mostly in the range of 0.68 to 0.90 for the original version. In previous studies internal consistency was good for internalizing problems (Cronbach’s alpha = 0.89) and excellent for externalizing (Cronbach’s alpha = 0.92) and total problems (Cronbach’s alpha = 0.95) [77]. The German version of the CBCL 6-18R [78] measures “external, internal and total problems” (superordinate scales) of children and adolescents between the age of 6 and 18 years. Internal consistency of the superordinate scales is reported as good to excellent (Cronbach’s alpha = 0.85 − 0.93) [78]. Our findings align since we also found internal consistency to be excellent for internalizing (Cronbach’s alpha = 0.94), externalizing (Cronbach’s alpha = 0.95) and total problem score (Cronbach’s alpha = 0.96).

### Emotion regulation strategies of parents and children

*Questionnaire to Assess Emotion Regulation in Children and Adolescents (FEEL-KJ).* The FEEL-KJ by Grob and Smolenski (2005) assesses ER strategies concerning fear, sadness, and anger among children and adolescents using a 5-point Likert scale (1 = “almost never” to 5 = “almost always”). While the original version consists of 90 items, we applied the self-report short version of the FEEL-KJ [31] in COMPI and COPWMI. It consists of 30 items in total, 14 items of which measure adaptive and 10 items maladaptive strategies. 6 items included in the questionnaire cannot be categorized clearly into either category. Each item of the short version integrates the three emotions of the original version into a superordinate emotional state (e.g., “If I am unhappy (sad, angry, anxious), I do not want to see anybody”). Older children were asked to complete the self-report version of the questionnaire on their own, younger children (preschool age up to 6 years old) provided their answers differently as they were unable to complete the questionnaire by themselves. The questions were read to the children by a researcher and they were asked to give their answer by placing a coin in boxes equivalent to the Likert scale used in the questionnaire. Those answers were then marked on the questionnaires Likert scales by the researcher. No reliabilities are reported for the short version of the self-report, yet the internal consistency for the original version of the self-report found in other studies is good for the higher-order scales adaptive (Cronbach’s alpha = 0.93) and maladaptive (Cronbach’s alpha = 0.82) ER strategies with two-week test-retest-reliabilities *r*_tt_ = 0.90 for adaptive and *r*_tt_ = 0.88 maladaptive ER strategies [79]. In our study sample the internal consistency was satisfactory (Cronbach’s alpha = 0.79).

*Questionnaire to Assess Emotion Regulation in Adults (FEEL-E).* To access adaptive and maladaptive ER strategies in parents of COPMI and COPWMI, we used the FEEL-E by Grob and Horowitz (2014). It is the adult version of the FEEL-KJ and consists of 90 items measuring six adaptive and maladaptive strategies using a 5-point Likert scale (1 = “almost never” to 5 = “almost always”). Both adaptive and maladaptive strategies can be displayed across emotions. In previous research Cronbach’s alpha ranges from 0.88 (maladaptive strategies) to 0.91 (adaptive strategies) for the higher-order scales. Test-retest-reliabilities after eight months are satisfactory for maladaptive and adaptive strategies (*r*_tt_ = 0.79) [80]. Sum scores for adaptive and maladaptive strategies were calculated across emotions for this study. We found internal consistencies to be satisfactory for anger (Cronbach’s alpha = 0.68), fear (Cronbach’s alpha = 0.62) and sadness (Cronbach’s alpha = 0.61).

### Eligibility measures

#### Socioeconomic status (SES)

To assess the SES, professional status and net household income were translated into numbers between 1 and 7 according to the scales used in the KiGGS study [74]. The mean of both values was computed. The KiGGS study, a large-scale health survey of children and adolescents in Germany, was used as a basis for calculating SES to ensure the comparability and validity of our socioeconomic status measurements within a well-established national framework.

### Diagnostic status of parents and children

*Diagnostic Interview for Mental Disorders (DIPS).* The DIPS [81] was used to assess whether parents of COPMI met the diagnostic criteria for study inclusion. The DIPS is a semi-structured diagnostic interview to determine mental disorders according to the DSM-5 [75, 81]. Parents of the COPWMI were only interviewed if the BSI was above the cut-off value (T_GSI_ ≥ 62). Previous studies report high inter-rater reliability using the instrument (0.72 < κ < 0.92) and test-retest reliabilities mostly in the range of 0.62 to 0.94 [82]. Diagnostic criteria as well as the severity of the diagnoses was assessed through judgement of a trained clinician.

*Diagnostic Interview for Mental Disorders During Childhood and Adolescence/Structured interview for Preschool Age (Kinder-DIPS/SIVA).* The diagnostic assessment of the children was conducted using the parent reports of the Kinder-DIPS [83] or SIVA [84]. The SIVA is a structured diagnostic interview for mental disorders for preschool ages according to ICD-10 and DC: 0–5. A translation table to DSM-5 diagnoses has been created for this study. The Kinder-DIPS is a structured diagnostic interview to determine mental disorders from age six to adulthood according to DSM-5. [85] report good to very good interrater reliabilities for the self- and parent-report of the Kinder-DIPS. For the SIVA good to very good interrater reliabilities for externalizing (κ > 0.82, > 91.9%), internaizing (κ > 0.72, 93.5%) and the xclusion of diagnoses (> 97%) are repoted [84]. Diagnostic interviews for COPMI were done by default. In the COPWMI group, parents were only interviewed if the value of total problems of the CBCL was above the cut-off value (T_CBCLSum_ ≥ 60).

### Statistical analysis

All statistical analyses were performed using SPSS version 27 [86]. Only complete datasets were included in the analyses while datasets with missing variables were excluded from further calculations. For the mediation analyses, the PROCESS tool was used [87]. The analytical strategy included preliminary analyses of possible differences between groups (COPMI vs. COPWMI) in the study variables according to demographic characteristics to address the need for potential confounding variables in the subsequent analyses.

Aim 1 was investigated with multivariate analyses of variance (MANCOVA) with group (COPMI vs. COPWMI) as between-subject variable and psychopathology measures (internalizing symptoms, externalizing symptoms, symptoms of general psychopathology) as within-subject’s variables. To prove the need of conducting a multivariate analysis correlations between outcome measures were computed. The test variables followed a multivariate normal distribution and observations were independent. The Pillai’s trace were used as statistics because the assumption of equality of covariance matrices was violated and Pillai’s statistics are robust. A descriptive discriminant analysis was carried out as the most frequently recommended and simplest multivariate post-hoc procedure for MANOVA [88]. Wilks’s lambda was used as statistic to test for statistical significance and to calculate the effect size. Standardized Discriminant Function Coefficients, Structure Coefficients, and Group Centroids were calculated to determine how each outcome variable contributed to group differences.

Aim 2 was investigated with two MANCOVAs with group (COPMI vs. COPWMI) as between-subject variable. One MANCOVA with parental maladaptive and adaptive ER strategies as within-subjects variables and one with child adaptive and maladaptive ER strategies as within-subjects variables. To prove the need of conducting a multivariate analysis correlations between outcome measures were computed. As the test variables followed a multivariate normal distribution, observations were independent and Box’s test for homogeneity of covariance matrices did not become significant, all assumptions for MANCOVA with child adaptive and maladaptive ER strategies as within-subjects variables were met. For parental ER strategies the assumption of equality of covariance matrices was violated. However, the Pillai’s statistics are robust. Wilks’s lambda was used as statistic to test for statistical significance and to calculate the effect size. Standardized Discriminant Function Coefficients, Structure Coefficients, and Group Centroids were calculated to determine how each outcome variable contributed to group differences.

Aim 3: Bivariate correlations were calculated to determine the relations between the study variables. Next, we calculated two sequential moderated mediations (with two mediators, model 87 of the PROCESS tool, [87]. One moderated mediation with internalizing and one with externalizing symptoms as outcome variable. In all moderated mediations, adaptive ER strategies of parents (mediator 1) and children (mediator 2) served as mediators and child maladaptive ER strategies as the moderator (between child adaptive ER strategies and child psychopathology measures). Indirect effects were estimated using the bootstrapping technique with 10,000 bootstrap samples and 95% BC confidence intervals. The moderated mediation model was determined to be significant if the 95% BC confidence interval did not contain zero. The relationship of all variables involved in the moderated mediation analysis was approximately linear, as assessed by visual inspection of the scatterplots after LOESS smoothing. Further, observations were independent. Since we used a robust method for the analyses, we dispense with checking normal distribution and heteroscedasticity. For statistical analyses an alpha level of 0.05 was applied and effect sizes (*η*_p_^2^) were calculated. Since the groups differed in SES (*p* < .001), this variable was included as a covariate in each analysis. We conducted post-hoc power analyses using G*Power 3.1 [89]. For the MANCOVA the achieved power (1-β) was found to be 0.97. For regression analysis it was calculated to be 0.99 and for the moderated mediation analysis, power was 0.95. These values indicate that our analyses were well-powered.

## Results

### Child psychopathology in COPMI versus COPWMI (Aim 1)

COPMI and COPWMI differed with respect to internalizing, externalizing and general symptoms of psychopathology with a large effect size (*V* = 0.13, *F*(3,190) = 9.66, *p* < .0001; *η*_p_^2^ = 0.132). The MANCOVA was followed up with discriminant analysis that revealed one discriminant function. It explained 100% of the variance, canonical *R*^2^ = 0.19. These discriminant function significantly differentiated the groups, Λ = 0.81, *χ*^2^(3) = 39.36, *p* < .0001. The correlation between outcomes and the discriminant function revealed that internalizing symptoms loaded highest onto the function (*r* = .75). However, externalizing (*r* = .55) and general psychopathological symptoms (*r* = .44) also loaded highly onto the function. For standardized coefficient see Table 2 and for Group Centroids Table 3. In addition, there was a positive association between the children’s and parents’ psychopathology (*r* = .36, *p* < .01). It must be noted that means of all psychopathology measures for both groups were within the normal range.

**Table 2:** Mean scores, Standard Deviations and standardized coefficients of psychopathology in children

	COPMI (*N* = 96)		COPWMI (*N* = 99)		Standardized coefficient
	*M*	*SD*	*M*	*SD*	
Child behavior checklist (*T* Scores)					
Internalizing symptoms	55.17	9.44	49.12	7.42	1.38
Externalizing symptoms	52.70	9.55	48.02	8.22	1.28
General psychopathology	51.76	8.73	48.32	7.74	- 1.69

SES was included as a covariate. Standardized Coefficients suggest large effects (small effect < 0.2, medium effect 0.2 to 0.5, large effect > 0.5)

**Table 3:** Group centroids of the three discriminant analyses

	Child Psychopathology	Child ER	Parent ER
**Group**			
COPMI	− 0.47	− 1.76	− 1.72
COPWMI	0.48	1.82	1.77

### Group differences in emotion regulation strategies (Aim2)

There was a medium effect of group (COPMI, COPWMI) on child’s adaptive and maladaptive ER strategies, Λ = 0.12, *F*(2,191) = 12.64, *p* < .0001; *η*_p_^2^ = 0.117 with the COPMI group scoring higher in both variables. The MANCOVA was followed up with discriminant analysis, which revealed one discriminant function. It explained 100% of the variance, canonical *R*^2^ = 0.22. These discriminant function significantly differentiated the groups, Λ = 0.78, *χ*^2^(2) = 48.88, *p* < .0001. The correlation between outcomes and the discriminant function revealed that both adaptive (*r* = .85) and maladaptive (*r* = .78) ER strategies loaded highly onto the function. For standardized coefficient see Table 4 and for Group Centroids Table 3.

**Table 4:** Quantile, Mean scores, Standard Deviations, and standardized coefficients of emotion regulation strategies in children and parents

	Total Sample (*N* = 195)		COPMI (*N* = 96)		COPWMI (*N* = 99)		Standardized coefficient
	Q_1_	Q_3_	*M*	*SD*	*M*	*SD*	
**Children**							
Adaptive ER strategies	20	47	39.41	13.03	28.67	10.46	0.62
Maladaptive ER strategies	11	31	23.66	8.30	16.66	7.60	0.58
**Parents**							
Adaptive ER strategies	86	113	104.83	17.76	96.08	18.88	0.29
Maladaptive ER strategies	44	105	106.46	18.36	46.78	17.26	1.00

Socioeconomic status was included as a covariate. Standardized Coefficients suggest medium to large effects (small effect < 0.2, medium effect 0.2 to 0.5, large effect > 0.5)

There was also a large effect of group on parental adaptive and maladaptive ER strategies (Λ = 0.64, *F*(2,191) = 169.33, *p* < .0001; *η*_p_^2^ = 0.639) with parents with mental illness demonstrating more adaptive and maladaptive ER strategies compared to healthy parents. The MANCOVA was followed up with discriminant analysis, which revealed one discriminant function. It explained 100% of the variance, canonical *R*^2^ = 0.76. These discriminant function significantly differentiated the groups, Λ = 0.25, *χ*^2^(2) = 270.09, *p* < .0001. The correlation between outcomes and the discriminant function revealed that adaptive loaded low (*r* = .14) and maladaptive highly onto the function (*r* = .96). For standardized coefficient see Table 4 and for Group Centroids Table 3.

Exploratory post hoc analyses showed that in children adaptive, *b* = 0.055, *z*^2^(1) = 2.116, *p* = .146, and maladaptive ER strategies, *b* = − 0.110, *z*^2^(1) = 3.053, *p* = .081, did not predict group membership (COPMI vs. COWMI) significantly if the interaction between the two, *b* = 0.006, *z*^2^(1) = 8.924, *p* = .003, was included in a logistic regression model, *χ*^2^(3) = 57.933, *p* < .0001, *R*^2^ = 0.343 (Nagelkerke). However, in adult’s adaptive ER strategies, *b* = 0.322, *z*^2^(1) = 12.275, *p* < .001, maladaptive ER strategies, *b* = 0.598, *z*^2^(1) = 14.854, *p* < .001, and the interaction between adaptive and maladaptive ER strategies, *b* = − 0.004, *z*^2^(1) = 11.412, *p* < .01, predicted the group membership (with mental illness vs. without mental illness) significantly (Nagelkerke *R*^2^ = 0.909).

### Moderated mediation analyses (Aim 3)

**Table 5:** Correlation matrix of study variables (total sample)

	Variables	1	2	3	4	5	6	7	8	9
	**Children**									
1	Adaptive ER	-								
2	Maladaptive ER	0.50**	-							
3	Internalising symptoms	0.12	0.31**	-						
4	Externalising symptoms	− 0.92	0.07	0.57**	-					
5	General psychopathology	− 0.06	0.15*	0.81**	0.90**	-				
	**Parents**									
6	Adaptive ER	0.23**	0.23**	− 0.02	− 0.05	− 0.05	-			
7	Maladaptive ER	0.38**	0.47**	0.41**	0.26**	0.27**	0.13	-		
8	Psychopathology	0.29**	0.29**	0.47**	0.34**	0.36**	− 0.12	0.70**	-	
9	SES	− 0.33**	− 0.31**	− 0.23**	− 0.13	− 0.13	− 0.23**	− 0.57**	− 0.45**	-

SES = Socioeconomic status

** *p* < .01

**Fig. 2:**
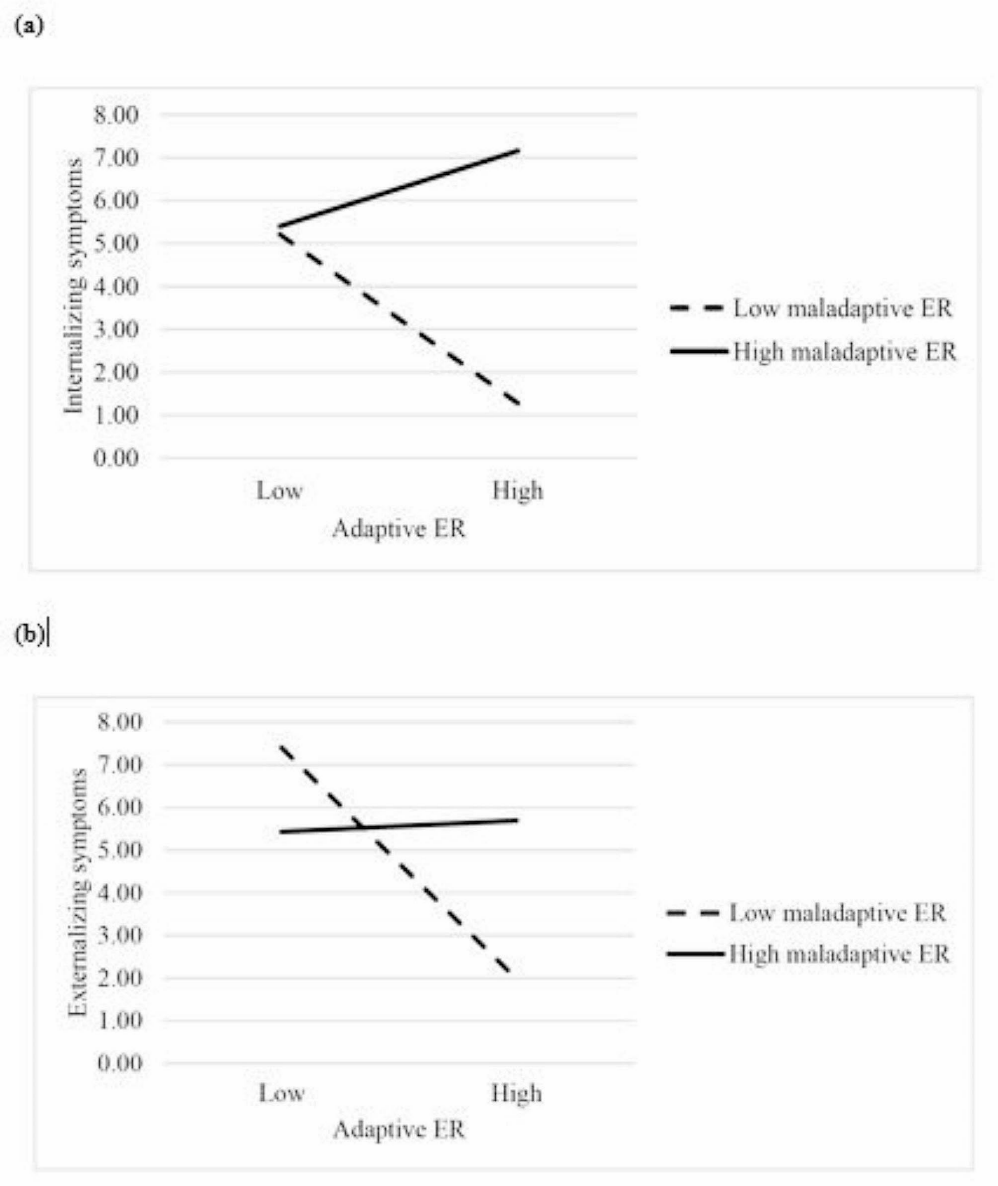
Interaction between adaptive and maladaptive ER strategies of children. High and low levels correspond to 1. and 3. quantile, respectively. Adaptive ER strategies of children have a negative association with child (**a**) internalizing and (**b**) externalizing symptoms *only* at low levels of maladaptive ER strategies of children

Table 5 presents bivariate correlations between dependent variables. The results of the moderated mediation were as follows:

1. Parental psychopathology positively predicted child internalizing symptoms, *b* = 0.073, 95% BCa CI [0.046, 0.101], *t* = 5.328, and child externalizing symptoms, *b* = 0.071, 95% BCa CI [0.040, 0.104], *t* = 4.396.
2. Furthermore, parental psychopathology negatively predicted parental adaptive ER strategies, *b* = − 0.184, 95% BCa CI [− 0.282, − 0.085], *t* = − 3.675.
3. Parental adaptive ER strategies, in turn, positively predicted child adaptive ER strategies, *b* = 0.155, 95% BCa CI [0.061, 0.250], *t* = 3.244.
4. There was a significant interaction between child adaptive and maladaptive ER strategies in the model with internalizing symptoms as outcome variable, *b* = 0.011, 95% BCa CI [0.005, 0.017], *t* = 3.588, as well as in the model with externalizing symptoms as outcome variable, *b* = 0.011, 95% BCa CI [0.004, 0.018], *t* = 3.033. Child adaptive ER strategies negatively predicted internalizing, *b* = − 0.145, 95% BCa CI [-0.222, − 0.069], *t* = − 3.754, and externalizing symptoms, *b* = − 0.201, 95% BCa CI [− 0.291, − 0.110], *t* = − 4.378, only when child maladaptive ER strategies were low. At high levels of maladaptive strategies, adaptive strategies were unrelated to internalizing, *b* = 0.065, 95% BCa CI [− 0.025, 0.154], *t* = 1.429, and externalizing symptoms, *b* = 0.010, 95% BCa CI [− 0.096, 0.116], *t* = 0.181. For demonstration of the interaction effects see Fig. 2.
5. The indirect effect of parent and child adaptive ER strategies on child internalizing and externalizing symptoms was significant at low levels (Q1) of child maladaptive ER strategies. It was not significant when child maladaptive ER strategies were high. Finally, the indirect effect of the moderated mediation was significant for child internalizing symptoms, *b* = − 0.0003, 95% BCa CI [− 0.0007, − 0.0001], and externalizing symptoms, *b* = − 0.0003, 95% BCa CI [− 0.0007, − 0.0001].

All reported relationships were in the predicted direction. For Regression Coefficients, Standard Errors, *p*-Values, and Model Summary Information see Table 6.

As an exploratory post hoc analysis we calculated a moderation analysis with maladaptive ER strategies of parents as moderator between parental adaptive ER strategies and psychopathology of parents. We found a negative association between adaptive ER strategies and psychopathology only when maladaptive ER strategies were high (Q_3_), *b* = − 0.735, 95% BCa CI [-0.956, − 0.515], *t* = -6.580, *p* < .001. When maladaptive ER strategies were low (Q_1_) the relationship was not significant, *b* = 0.018, 95% BCa CI [-0.201, 0.238], *t* = 0.165, *p* = .869. We further calculated two moderation analyses with maladaptive ER strategies of children as moderator between children adaptive ER strategies and psychopathological symptoms of children, one analysis with younger children (< 10 years) and one with older children (≥ 10 years) to test the age effect. We found that the interaction explained more variance in psychopathological symptoms of children when the children were younger, Δ*R*^2^ = 8.30, *F*(1,88) = 8.211, *p* = .005, than when they were older, Δ*R*^2^ = 4.81, *F*(1,99) = 5.682, *p* = .019.

**Table 6:** Regression coefficients, Standard Errors, and Model Summary Information for Child Internalizing and externalizing symptoms

	M_1_ (parent adaptive ER)			M_2_ (child adaptive ER)			Y (internalizing sympt.)			Y (externalizing sympt.)		
	*B*	*SE*	*p*	*B*	*SE*	*p*	*B*	*SE*	*p*	*B*	*SE*	*p*
*X* (parent psychopathology)	− 0.184	0.050	< 0.001	0.111	0.034	0.001	0.073	0.014	< 0.0001	0.071	0.016	< 0.0001
*M*_*1*_ (parent adaptive ER)	-	-	-	0.155	0.048	0.001	− 0.003	0.019	0.891	0.007	0.023	0.741
*M*_*2*_ (child adaptive ER)	-	-	-	-	-	-	− 0.263	0.065	< 0.001	− 0.318	0.077	< 0.0001
*W* (child maladaptive ER)	-	-	-	-	-	-	− 0.204	0.110	0.064	− 0.315	0.130	0.016
*M*_*2*_ x *W*	-	-	-	-	-	-	0.011	0.003	< 0.001	0.011	0.004	0.003
*Covariate* (SES)	− 5.754	1.244	< 0.0001	− 1.869	0.870	0.033	0.008	0.336	0.982	− 0.050	0.406	0.902
Constant	136.180	7.489	< 0.0001	25.501	0.820	0.002	8.646	3.728	0.022	12.322	4.416	0.006
	*R*^2^ = 0.114			*R*^2^ = 0.179			*R*^2^ = 0.314			*R*^2^ = 0.196		
	*F*(2,192) = 12.295***			*F*(3,191) = 13.901***			*F*(6,188) = 14.43***			*F*(6,188) = 7.647***		

Sympt. = symptoms, SES = Socioeconomic status, *** *p* <. 0001

## Discussion

In this study, we sought to investigate ER strategies in COPMI and COPWMI as a factor associated with psychopathology and thus representing a potential target for preventive interventions for COPMI. Our first aim was to confirm that COPMI have a higher risk of psychopathology compared to COPWMI [7, 8, 93, 94], which was supported by our findings, showing higher psychopathological symptoms in COPMI. Despite this, the mean T-Scores for internalizing, externalizing, and general psychopathology were within the normal range for both groups. Comparing our results with Loechner et al. (2020), our COPMI sample had lower internalizing and general psychopathology scores but similar externalizing scores. Wiegand-Grefe et al. (2011) reported higher CBCL scores for COPMI, yet still within normal ranges. The differences in psychopathological symptoms between our groups were smaller than those reported by Loechner et al. (2020), and fewer COPMI in our sample fell into subclinical or clinical ranges compared to Wiegand-Grefe et al. (2011). The lower levels of psychopathology observed in our COPMI sample compared to other studies can be attributed to our exclusion criteria and recruitment settings. By excluding children with severe impairments requiring comprehensive treatment and focusing on parents from outpatient clinics, we selected a sample with relatively milder symptoms.

The second aim was to compare ER strategies in COPMI and COPWMI and their parents. We hypothesized more maladaptive and fewer adaptive ER strategies in COPMI and their parents. This was confirmed only for maladaptive strategies, with COPMI and their parents showing increased maladaptive ER strategies, consistent with previous studies linking maladaptive ER strategies to higher psychopathology [14, 15, 61–63]. Given the significant impact of parents on their child’s acquisition and application of ER strategies via emotion socialization [37–42], it makes sense that children have more (or less) ER strategies when their parents have more (or less) ER strategies. In fact, our results show that this is true for maladaptive as well as adaptive ER strategies. In contrast to previous empirical data [7, 14, 64, 65], COPMI and their parents did not show fewer but more adaptive ER strategies than COPWMI and their parents. This could be due to their lower psychopathology levels compared to other studies [7, 90], as adaptive ER strategies are associated with lower psychopathology [14]. In parents, maladaptive ER strategies explained more variance than adaptive ones, aligning with past findings [14]. However, in children, adaptive strategies explained almost as much variance as maladaptive ones. Our study used self-report measures across a wide age range, unlike many previous studies that used observational methods in younger children [64–66, 91]. This difference in methods and age ranges might contribute to the divergent results. ER strategies develop significantly from infancy to adulthood, with emotional awareness and access to ER strategies increasing with age [92]. COPMI may use more overall ER strategies due to the stress from their parents’ mental illness.

Third, we found that the indirect effect of parent and child adaptive ER strategies on child internalizing and externalizing symptoms was only significant at low levels of child maladaptive ER strategies. In accordance with the interference hypothesis [29], child adaptive ER strategies seem to have only a protective effect for developing psychopathological symptoms in children if child maladaptive ER strategies are low. This finding is contrary to most of the previous studies that confirmed the compensatory hypothesis [29] in healthy adults [29, 70, 71]. To our knowledge, this is the first published study examining the interactive effect of adaptive and maladaptive ER strategies in children on psychopathology. One possible and apparent explanation for the differing results is the age of the investigated sample. The interaction may take a different shape in children than in adults. In fact, in adults we found a negative association between adaptive ER strategies and psychopathology only when maladaptive ER strategies were high. In turn, this finding is consistent with previous ones supporting the compensatory hypothesis [29, 70, 71]. This might suggest that the effectiveness of adaptive ER strategies depends on other strategies, namely the maladaptive ER strategies, of an individual. The compensatory hypothesis suggests that the use of adaptive strategies may be most beneficial for those individuals who also use maladaptive strategies frequently. This might suggest that a rich repertoire of ER strategies, along with a flexible and appropriate use of them, is more important than specific ER strategies [71]. In contrast to this, in children the use of maladaptive ER strategies might interfere with the effectiveness of adaptive ER strategies. Children having both high maladaptive and adaptive ER strategies might not able to select the appropriate ER strategy in accord with the environmental demand. As a consequence, children might fail to experience the benefits that are associated with adaptive ER strategies [95] and have increased psychopathological symptoms. The assumption of an age effect is supported from the developmental psychological perspective. Between the ages of 7 and 12, an emotional awareness develops. Afterwards, children can also consciously perceive various ER strategies. This conscious perception can in turn be used to control emotions in adolescence and to become aware of the effectiveness of the strategies used in young adulthood [92]. In fact, we found that the interaction of the ER strategies explained more variance in the psychopathological symptoms in younger children (< 10 years) than in older children (≥ 10 years). In this respect, it will be essential for future investigations to distinguish between younger and older children as well as adults, to replicate these findings and implement experimental designs to reveal the underlying mechanisms of the interfering effect of maladaptive ER strategies.

### Strengths, implications and limitations

The main strength of this study is modelling ER as a factor associated with psychopathology and investigating both parental and child ER in a sample with a wide range of psychopathology. In this way, it integrates transgenerational transmission of ER and psychopathology in one model and thus extends previous literature that is limited to single relationships. For instance, parental adaptive ER strategies have been negatively related to psychopathology in adults [14, 15] and adaptive ER strategies of parents were positively associated with adaptive ER strategies in children [43, 44, 47]. However, no prior study has examined each step of this pathway in one sample, such that moderated sequential mediation and indirect pathways between parental and child psychopathology could be pursued. The significant indirect effect of the moderated mediation suggests that the transgenerational transmission of adaptive ER strategies and the interaction between adaptive and maladaptive ER strategies of children may represent one pathway by which parental psychopathology affects child outcomes. Another strength is the clinical subsample in this study which enables stronger conclusions about the trans-generational effect of parental mental illness than studies based on community samples. Further, the present study is the first one to examine ER in a clinical sample not limited to certain mental disorders like depression. This allows us to expand the findings on other mental disorders and the conclusion that ER seems to be a mechanism relevant in TTMD in various mental disorders. However, it has to be mentioned that the majority of our sample consisted of parents with internalizing disorders and subgroups for specific disorders were too small to conduct further analyses in relation to the parental disorder type. This should be taken into consideration in further studies, to expand our findings onto even more heterogeneous COPMI groups. The number of clinically-relevant instruments including the clinical interviews conducted with both mentally ill parents and COPMI, the large sample size and the representativeness of the clinical sample should also be positively emphasized. Beyond, this study is one of few studies to compare ER in COPMI and COPWMI directly.Aside from the strengths, several limitations need to be mentioned. One limitation of the study is that parents reported the psychopathology for themselves and their children. Parent-ratings alone have been shown to be less valid for children’s internalizing symptoms but more valid for externalizing symptoms [85, 96]. Furthermore, mentally ill parents may overestimate the psychopathology of their children due to their own psychopathology. However, for ER parent- and child ratings are available and reveal significant medium (adaptive ER: *r* =.386) to high (maladaptive ER: *r* =.475) correlations indicating sufficient correspondence between parents and children in this sample. Another limitation is that our analyzes are only based on questionnaires. Previous literature recommended that ER should be studied as a multicomponent process including multiple types of measurement (e.g. self-report, behavior coding measure) [34]. This should be taken into account in future studies. Moreover, in future studies objective measurements, like psychophysiological measures, should be included to possibly solve the problem of inconsistent measurement (and results) of ER across studies. A final limitation of the study is that the data are cross-sectional rather than longitudinal and therefore do not allow causal interpretations to be drawn about ER as a factor, which prospectively predicts the onset of a mental disorder in COPMI. In order to capture developmental risks and model resilience for mental illness, longitudinal studies are needed. We are currently collecting data of further measurement points on the participants of the COPMI group in this study. This would allow us to address these questions. If prospective longitudinal research will support the present findings, they may have important implications for developing prevention and intervention programs for COPMI and thus interrupt the TTMD. Another construct further research should take into account in the context of ER in COPMI is parent-child-attachment. Parent-child-attachment and ER abilities in children are closely related [48–52] and both ER as well as parent-child-attachment have been found to be impaired in parents with a mental disorder and their children. Therefore, future research would profit from assessing parent-child-attachment additionally to ER in a large and diverse COPMI sample to assess the relation between both constructs and their role in TTMD. Our results highlight important clinical implications. First, they indicate that COPMI should receive preventive training in ER since it can be assumed that ER is almost always affected in patients with mental illness. Second, in contrast to recommendations to enhance adaptive ER strategies during treatment [97], it may be important to reduce the use of maladaptive ER strategies in COPMI. According to our results of the moderated mediation analysis, a reduction in the use of maladaptive ER strategies could contribute to an interruption of the transgenerational transmission of psychopathological symptoms by enabling the beneficial and protective effects of adaptive ER strategies. Preventive interventions for COPMI should therefore potentially include ER training that focus rather on reducing maladaptive ER strategies than promoting adaptive ER strategies. It could also be helpful to work metacognitively with COPMI on the use of ER strategies. In this way, they could learn which strategies are effective for them under which conditions. Due to the different levels of the psychopathological symptoms and ER strategies in COPMI, it could also be useful to assess these levels beforehand in order to adapt the training individually.

Our findings suggest that the severity of parental mental illness plays a critical role in the development of ER strategies in COPMI. In cases of milder parental mental illness, children may develop a wider range of both adaptive and maladaptive ER strategies. However, severe mental illness in parents might disrupt this balance, leading primarily to maladaptive strategies. This highlights the need for tailored interventions in clinical and community settings, focusing on reducing maladaptive ER strategies and enhancing adaptive ones, especially in families affected by severe mental illness.

## Conclusions

Taken together, our study demonstrates that COPMI show significantly higher levels of psychopathology compared to COPWMI. Both COPMI and their parents exhibit more maladaptive and adaptive ER strategies. Importantly, the study reveals that adaptive ER strategies in children only mitigate psychopathological symptoms when maladaptive ER strategies are low. These findings suggest that the interaction between adaptive and maladaptive ER strategies plays a crucial role in the transgenerational transmission of psychopathology and therefore underscores the necessity for comprehensive and tailored approaches in understanding and addressing the TTMD.

## Data Availability

Data not available due to legal restrictions.

## Abbreviations

COPMI: Children of parents with mental illness
COPWMI: Children of parents without mental illness
TTMD: Transgenerational transmission of mental disorders
ER: Emotion regulation
